# Prediction of the lower serum anti-Müllerian hormone threshold for ovarian stimulation prior to in-vitro fertilization using the Elecsys® AMH assay: a prospective observational study

**DOI:** 10.1186/s12958-019-0452-4

**Published:** 2019-01-11

**Authors:** A. G. Grynnerup, K. Løssl, F. Pilsgaard, S. A. Lunding, M. Storgaard, J. W. Bogstad, L. Prætorius, A. Zedeler, L. Bungum, A. Nyboe Andersen, A. Pinborg

**Affiliations:** 1 Fertility Clinic, Copenhagen University Hospital Hvidovre, Kettegård Allé 30, DK-2650 Hvidovre, Denmark; 2 Fertility Clinic, Rigshospitalet Copenhagen University Hospital, Blegdamsvej 9, DK-2100 Copenhagen, Denmark; 3 Fertility Clinic, Copenhagen University Hospital Herlev, Herlev ringvej 75, DK-2730 Herlev, Denmark

**Keywords:** In vitro fertilization (IVF), Ovarian stimulation, Anti-Müllerian hormone (AMH), Poor ovarian response, Receiver operating characteristics (ROC) curve analysis

## Abstract

**Background:**

In assisted reproductive technology, prediction of treatment failure remains a great challenge. The development of more sensitive assays for measuring anti-Müllerian hormone (AMH) has allowed for the possibility to investigate if a lower threshold of AMH can be established predicting very limited or no response to maximal ovarian stimulation.

**Methods:**

A prospective observational multicenter study of 107 women, < 40 years of age with regular menstrual cycle and serum AMH levels ≤ 12 pmol/L, treated with 300 IU/day of HP-hMG in a GnRH-antagonist protocol. AMH was measured before treatment start using the Elecsys® AMH assay by Roche Diagnostics. The ability of AMH to predict follicular development and ovarian response was assessed by receiver operating characteristics (ROC). Furthermore, the relationship between AMH at start of stimulation and cycle outcome was investigated using multivariate logistic regression analysis.

**Results:**

Five out of 107 cycles (4.7%) were cancelled due to lack of follicular development and 60/107 (56%) women did not reach the classical hCG criteria for ovulation induction (≥ 3 follicles of ≥17 mm). An AMH threshold of 4 pmol/L predicted failure to reach the classical hCG criteria with 89% specificity and 53% sensitivity and an area under the curve (AUC) of 0.76 (95% CI 0.66–0.85). AMH predicted cycle cancellation due to lack of follicular development, using a cut-off value of 1.5 pmol/L, with a specificity of 96% and sensitivity of 80% (AUC = 0.92, 95% CI 0.79–1.00). A single-unit increase in AMH was associated with a 29% decrease in odds of failure to reach the classical hCG criteria (OR 0.71 95% CI 0.59–0.85, *p* < 0.01). The lowest AMH value compatible with a live birth was 1.3 pmol/L.

**Conclusions:**

Among women with a limited ovarian reserve, pre-treatment serum AMH levels significantly predicted failure to reach the classical hCG triggering criteria and predicted lack of follicular development using a new sensitive assay, but AMH was not suitable for withholding fertility treatment, as even very low levels were associated with live births.

**Trial registration:**

Not relevant

## Background

Anti-Müllerian Hormone (AMH) is a glycoprotein secreted from the granulosa cells of preantral and antral follicles [1], and has shown to be a good predictor of the response to ovarian stimulation for assisted reproductive technology (ART) [2]. AMH has therefore gained widely use in the clinical practice of fertility clinics and is considered as valid as antral follicle count (AFC), but has the advantage of less inter observer variability [3].

Poor ovarian reserve affects 5–10% of patients undergoing ART depending on the average female age of the infertile population [4], and is associated with decreased yield of oocytes, cycle cancellation and a reduced probability of pregnancy [5]. One of the clinical challenges regarding patients with poor ovarian reserve is to identify the patients in whom the ovarian reserve is below the lower threshold for ovarian stimulation. In other words, to identify those women with a severely affected ovarian reserve, where maximal gonadotrophin stimulation will result in less than 3 mature follicles, which define the classical criteria for triggering of ovulation with hCG, and thus result in cycle cancellation.

Several different studies have addressed the issue of predicting outcome in poor prognosis patients [6–10]. However, various AMH assays and the lack of an international reference standard for AMH has made it difficult to compare data from different clinical settings. The most extensively used AMH assay is the Beckman-Coulter AMH Gen II assay. However, concern has been raised regarding the reliability of the assay as within-subject variability and complement interference has been documented [11]. Furthermore, the original Beckman-Coulter AMH Gen II assay could not detect levels of AMH < 3 pmol/L. Patients with AMH values below the detection level have demonstrated acceptable live births rates, as assessed by Seifer et al. in a SART database study of over 5000 cycles reporting of live-birth rate of 9.5% in women with AMH levels below the detection limit [6]. Thus, the lower threshold of AMH, where patients will have no ovarian response, has not yet been established, and it could be suspected that the true threshold for ovarian stimulation lies beneath the limit of detection by the AMH Gen II assay.

New AMH assays have been developed including the fully automated Elecsys® AMH Immunoassay (Roche Diagnostics International Ltd) and the picoAMH assay (Ansh Labs), both of which can detect levels of AMH < 3 pmol/L [12, 13]. Burks et al. used the picoAMH assay in a retrospective case-control study (*n* = 48) aiming to identify the lower threshold for ovarian stimulation [14]. They found that the assay performed well in predicting failure to achieve oocyte retrieval, however clinical pregnancy was observed in women with very low AMH levels (1.0 pmol/L). Unlike the Elecsys® assay, the picoAMH assay is not automated. Furthermore, it was designed for the detection of very low AMH levels, and thus the standard curve is centered around very low values (standard curve range 1–746 pg/mL), resulting in the majority of samples requiring dilution before utility of the assay [15].

Compared with the AMH Gen II assay, the Elecsys® AMH assay have demonstrated lower analytical variability and closer correlation with AFC in the lower range of AMH values [16, 17]. These differences could be explained by improved analytical features such as lower limit of quantification and maximum imprecision as compared with the AMH Gen II assay [17]. The enhanced performance in the lower AMH concentration range by the new assays may enable a better guidance and counsel of women with weak AMH production. Whether it is possible to detect a minimum AMH cut-off value for ovarian stimulation using a more sensitive assay with a lower detection level is unknown. In this study the pre-treatment AMH levels were measured prospectively in women with limited ovarian reserve treated in a maximal gonadotrophin stimulation protocol, to investigate if the use of the Elecsys® AMH assay with a lower detection threshold than the old assays, can predict failure to reach the classical criteria for hCG trigger or failure to develop at least one mature follicle. Thereby improving the counselling of couples regarding their realistic chances of success in ART.

## Materials and methods

### Study design and aim

Prospective observational multicenter cohort study performed in three public fertility clinics in Denmark with the aim of investigating if serum AMH measured with the new automated Elecsys® AMH assay could predict failure to reach the classical criteria for hCG trigger or failure to develop a single mature follicle.

### Patients

Women between 18 and 40 years of age and BMI < 35, who were referred for IVF/ICSI treatment with a regular menstrual cycle between 24 and 35 days and a pre-treatment AMH ≤ 12 pmol/L, measured with the automated Elecsys® AMH assay, were included in the study from December 2015 to April 2017. All included women had both ovaries and gave informed consent in writing. Exclusion criteria were: Endometriosis stage III-IV, severe comorbidity (i.e. IDDM, NIDDM, gastrointestinal, cardiovascular, pulmonary, liver or kidney diseases), dysregulation of thyroid disease, not Danish or English speaking, ovarian cyst at start of stimulation, and previous inclusion in the study.

The AMH cut-off value for inclusion of 12 pmol/L corresponds to 15 pmol/L measured with the former manual ELISA assay (AMH Gen II assay) as values are expected to be 20% lower when measured with the Elecsys® AMH assay [17]. The cut-off value was chosen on the basis of the study by Yates et al. [18] in which a cut-off of 15 pmol/L, measured with the AMH Gen II assay, was used to identify women with limited ovarian reserve.

### Clinical protocol and data collection

All were treated in a GnRH-antagonist protocol with a fixed dose of 300 IE HP-hMG with fixed daily GnRH antagonist 0.25 mg/day added from stimulation day 6. Triggering of ovulation by hCG was done when follicles reached a size ≥17 mm, and any patient with at least one follicle ≥17 mm was offered hCG trigger and oocyte retrieval. Oocyte retrieval was performed 36 ± 2 h after hCG administration. Oocytes were fertilised by either in vitro fertilization (IVF) or intracytoplasmic sperm injection (ICSI) and embryos were cultured individually according to standard procedures. Single Day 2 embryo transfer was performed and, if available, surplus high-quality embryos were cryopreserved on either Day 2 or 5. A serum hCG test was performed two weeks after embryo transfer. Clinical pregnancy was confirmed by transvaginal ultrasound 5–6 weeks after embryo transfer. A positive hCG test was defined as a serum hCG level of more than 5 IU/L two weeks after embryo transfer, and ongoing pregnancy was defined as a foetus with foetal heartbeat at gestational week 7–8.

Data collection was performed at baseline (cycle day 2–3), stimulation day 6, and on day of hCG triggering. Blood samples were collected, and transvaginal ultrasonography was performed on all three occasions. Size and number of antral follicles at all occasions were registered. Serum AMH concentrations were measured using the fully automated Elecsys® AMH assay from Roche Diagnostics on the Cobas e 601 analyzer in accordance with the manufacturer protocols. The assays limit of detection was 0.07 pmol/L and limit of quantitation was 0.21 pmol/L.

### Outcome measures

The primary endpoint was the lower serum AMH threshold that best predicted failure to reach the classical hCG criteria (at least 3 follicles of ≥ 17 mm on day of hCG administration). The secondary endpoints and outcome variables were AMH cut-off values predicting cycle cancellation due to lack of follicular development, embryo transfer, low oocyte yield (≤ 3 oocytes), positive hCG test as well as ongoing pregnancy and live births. For analytic purposes, low oocyte yield was defined as ≤3 oocytes, as used in the Bologna criteria for poor ovarian response [19].

### Statistical analysis

Continuous data are presented in median and interquartile range (IQR) and categorical data are presented in frequencies and percentages. Receiver operator characteristic (ROC) curve analyses for AMH and AFC predicting failure to reach the classical hCG criteria, cycle cancellation, embryo transfer, low oocyte yield, positive hCG test and live birth were calculated. Youden’s Index (sensitivity + specificity − 1) were calculated to identify the optimal cut-off points, however to provide the most applicable cut-off values for clinical practice, cut-off values with high specificity were preferred when assessing failure to reach the classical hCG criteria, cycle cancellation due to lack of follicular growth, and low oocyte yield, while high sensitivity was preferred when assessing embryo transfer, positive hCG test and live birth. ROC curves for AMH and AFC were compared using χ^2^-test.

The ability of baseline serum AMH and AFC to predict failure to reach the classical hCG criteria was further evaluated with univariate logistic regression analysis along with other explanatory parameters: FSH, age, BMI, cycle length, previous no. of ART cycles, parity and duration of infertility. A multivariate logistic regression model was then performed including the explanatory parameters found to be significantly associated with failure to reach the classical hCG criteria in the univariate logistic regression analysis.

Sample size calculation was performed prior to initiating the study. To detect an area under the curve (AUC) of 0.65 for AMH on predicting failure to reach the classical hCG criteria, a sample size of 100 patients would be necessary to achieve at least 80% power with a 5% one-sided significance level. This was based on the assumption that the proportion of patients failing to reach the classical hCG criteria was within the range 0.3–0.7, as would be expected as only patients with AMH ≤ 12 pmol/L were included.

## Results

**Fig. 1:**
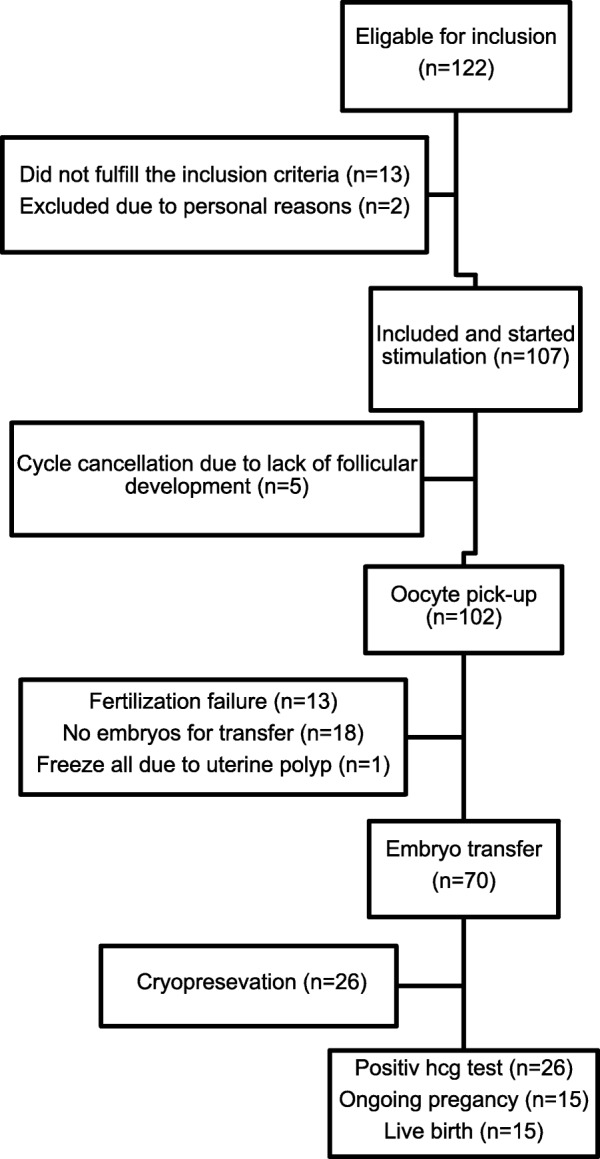
Flow-chart of included patients

All 107 included women started ovarian stimulation with hMG (Fig. 1). Patients characteristics are depicted in Table 1. The median age (IQR) was 36 (34–38) years, cycle length 26 (24–27) days and AMH 5 (3.3–8.3) pmol/L. In total sixty women (56% per started cycle) did not reach the classical hCG triggering criteria of at least 3 follicles ≥ 17 mm at the day of hCG administration and five women (4.7% per started cycle) had their cycle cancelled due to lack of follicular development (Table 2). Among the women that failed to reach the classical hCG criteria, 6 out of 60 (10%) achieved an ongoing pregnancy and live birth, compared with 9 out of 47 (19%) in the group that reached the classical hCG criteria. No cycles were converted from IVF/ICSI to intrauterine insemination. None of the participants had their cycle cancelled or had GnRH agonist triggering due to risk of ovarian hyperstimulation syndrome (OHSS).

**Table 1:** Patient demographics, fertility characteristics and baseline endocrinology

Demographics	Median (IQR) or *n* (%)
Age, years	36 (34–38)
BMI, kg/m^2^	22.9 (20.4–25.6)
Cycle length, days	26 (24–27)
Smoking, *n* (%)	8 (7.4%)
Fertility characteristics	
Duration of infertility, months	24 (18–36)
1. ART cycle, *n* (%)	75 (70)
2. ART cycle, *n* (%)	17 (16)
3. ART cycle, *n* (%)	13 (12)
> 3. ART cycle, *n* (%)	2 (2)
Etiology of infertility, *n* (%)	
Anovulation	2 (2%)
Tubal factor	16 (15%)
Endometriosis	4 (4%)
Male factor	42 (40%)
Unexplained	25 (24%)
Other	33 (31%)
Baseline endocrinology and ultrasound (cycle day 2–3)	
AMH, pmol/L	5.0 (3.3–8.3)
FSH, IU/L	10.0 (8.0–13.2)
Progesterone, nmol/L	1.3 (1.0–2.0)
Estradiol, nmol/L	0.12 (0.09–0.17)
AFC, no. of follicles	8 (5–11)

Continuous variables are presented in median (interquartile range (IQR)), and categorical data are presented in number of occurrence (frequencies in percent)

*BMI* body mass index, *ART* assisted reproductive technologies, *AMH* anti-Müllerian hormone, *FSH* follicle stimulating hormone, *AFC* antral follicle count

**Table 2:** Cycle characteristics and outcome after ovarian stimulation and IVF/ICSI

	Per started cycle (*n* = 107)
Cycles with ovarian stimulation, *n* (%)	107 (100%)
Stimulation days, median (IQR)	8 (7–10)
Cycles cancelled, *n* (%)	5 (4.7%)
Cycles reaching the classical hCG criteria, *n* (%)	47 (44%)
Oocyte retrieval, *n* (%)	102 (95%)
No. of oocytes retrieved, median (IQR)	2 (2–3)
Fertilization rate in %, median (IQR)	66.7 (41–100)
Embryo transfer, *n* (%)	70 (65%)
Embryo cryopreservation, *n* (%)	28 (26%)
Positive hCG test, *n* (%)	26 (24%)
Ongoing pregnancy, *n* (%)	15 (14%)
Live birth, *n* (%)	15 (14%)
	Per embryo transfer (*n* = 70)
Positive hCG test, *n* (%)	26 (37%)
Ongoing pregnancy, *n* (%)	15 (21%)
Live birth, *n* (%)	15 (21%)

*IQR* interquartile range, *IVF* in vitro fertilization, *ICSI* intracytoplasmic sperm injection, *hCG* human chorionic gonadotropin

### Predicting follicular development

**Fig. 2:**
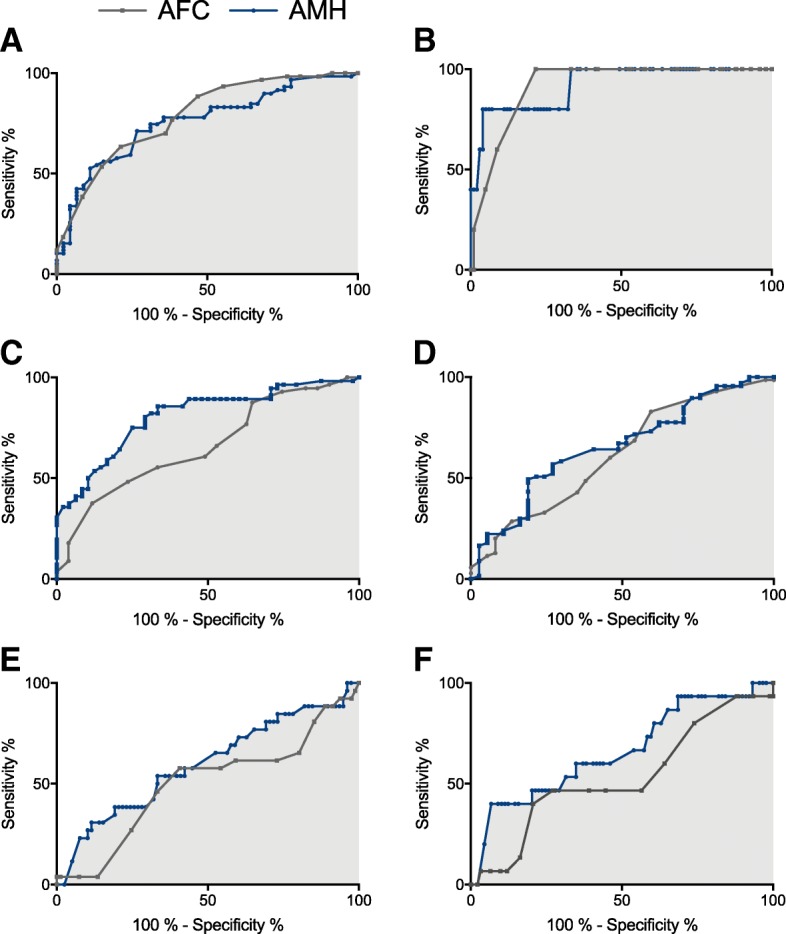
Receiver Operating Characteristic (ROC) curves for anti-Müllerian hormone (AMH) and antral follicle count (AFC) for predicting **a**) Failure to reach the classical hCG criteria; **b**) Cycle cancellation; **c**) Low oocyte yield (≤ 3 oocytes); **d**) Embryo transfer; **e**) positive hCG test; **f**) Live birth

ROC-curves depicting the ability of AMH and AFC to predict failure to reach the classical hCG criteria are presented in Fig. 2a and results from the ROC-curve analysis are presented in Table 3. The AUC was 0.76 (95% confidence interval (CI) 0.66–0.85) for AMH. There was no statistically significant difference between the two ROC-curves for AMH and AFC (*p* = 0.53). Including both AFC and AMH in the model provided an AUC of 0.80 (95% CI 0.72–0.89). The optimal AMH cut-off value was 4.0 pmol/L providing a specificity of 89% and sensitivity of 53% (false positive rate (FPR): 11%, false negative rate (FNR): 47%). Given the prevalence of failure to reach the classical hCG criteria in this population (56%), the positive predictive value at this cut-off point was 86% (the proportion of positive test results that were true positive), while the negative predictive value was 60% (the proportion of negative test results that were true negative). The lowest serum AMH level observed among women who did reach the classical hCG criteria was 1.30 pmol/L.

**Table 3:** Receiver operator characteristics curve analysis

	AUC	95% CI	*p*-value*	Optimal cut-off value	Sensitivity	Specificity
Failure to reach classical hCG criteria (*n* = 60)						
AMH	0.76^a^	0.66-0.85	< 0.001	4.0 pmol/L	53%	89%
AFC	0.79^a^	0.70-0.88	< 0.001	7	63%	78%
Model AFC & AMH	0.80^a^	0.72–0.89	< 0.001			
Cycle cancellation (*n* = 5)						
AMH	0.92^a^	0.80-1.00	< 0.001	1.5 pmol/L	80%	96%
AFC	0.92^a^	0.86-0.99	< 0.001	4	60%	91%
Model AFC & AMH	0.95^a^	0.86–1.00	< 0.001			
Low oocyte yield (*n* = 57)						
AMH	0.81^b^	0.73-0.89	< 0.001	4.0 pmol/L	54%	88%
AFC	0.68^b^	0.57-0.78	< 0.001	5	38%	88%
Model AFC & AMH	0.81^b^	0.71–0.88	< 0.001			
Embryo transfer (*n* = 70)						
AMH	0.66^a^	0.55-0.77	0.005	4.2 pmol/L	72%	46%
AFC	0.63^a^	0.51-0.74	0.030	6	83%	41%
Model AFC & AMH	0.65^a^	0.54–0.76	0.009			
Positive hCG test (*n* = 26)						
AMH	0.60^a^	0.46-0.73	0.154	4.4 pmol/L	73%	40%
AFC	0.50^a^	0.36-0.63	0.952	6	73%	25%
Model AFC & AMH	0.61^a^	0.48–0.75	0.087			
Live birth (*n* = 15)						
AMH	0.66^a^	0.50-0.82	0.047	3.8 pmol/L	93%	31%
AFC	0.53^a^	0.36-0.70	0.750	6	80%	26%
Model AFC & AMH	0.70^a^	0.55–0.85	0.008			

AUC: area under the curve; CI: confidence interval; AMH: anti-Müllerian hormone; AFC: antral follicle count

*Null-hypothesis: true AUC = 0.5

^a^No statistically significant difference between AFC and AMH ROC-curves

^b^Statistical significant difference between AFC and AMH ROC-curves *p* = 0.004

Univariate and multivariate logistic regression analysis of explanatory parameters in predicting failure to reach the classical hCG criteria are presented in Table 4. Besides AMH, AFC, age, cycle length, and number of previous ART cycles were statistically associated with failure to reach the classical hCG criteria. When adding these parameters to a multivariate logistic regression model, a single-unit increase in AMH was associated with a 29% decrease in odds of failing to reach the criteria (adjusted odds ratio (aOR) 0.71, 95% CI 0.59–0.85, *p* = 0.0001).

**Table 4:** Univariate and multivariate logistic regression analysis for predicting failure to reach the classical hCG criteria

	OR	95% CI	*p* value
Univariate analysis			
AMH	0.72	0.62–0.85	<.0001
AFC	0.72	0.62–0.83	<.0001
FSH	1.07	0.98–1.17	0.12
Age	1.14	1.01–1.29	0.04
BMI	1.02	0.92–1.13	0.73
Cycle length	0.83	0.70–0.98	0.02
No. of prev. ART cycles	0.50	0.29–0.87	0.02
Parity	0.80	0.34–1.91	0.62
Duration of infertility	1.01	0.99–1.03	0.19
Multivariate analysis with AMH			
AMH	0.71	0.59–0.85	0.0001
Age	1.20	1.03–1.39	0.02
Cycle length	0.84	0.69–1.02	0.07
No. of prev. ART cycles	0.46	0.22–0.93	0.03
Multivariate analysis with AFC			
AFC	0.69	0.59–0.82	0.0001
Age	1.14	0.98–1.34	0.10
Cycle length	0.77	0.62–0.96	0.02
No. of prev. ART cycles	0.38	0.19–0.77	0.01

*OR* odds ratio, *CI* confidence interval, *AMH* anti-Müllerian hormone, *AFC* antral follicle count, *FSH* follicle stimulating hormone, *ART* Assisted Reproductive Technology, *hCG* human chorionic gonadotropin

AMH and AFC both performed well in predicting cycle cancellation due to lack of follicular development with an AUC of 0.92 (95% CI 0.86–1.00) and 0.92 (95% CI 0.86–0.99), respectively (Fig. 2b and Table 3). A serum AMH cut-off value of 1.5 pmol/L predicted cycle cancellation with a specificity of 96% and a sensitivity of 80%.

### Predicting embryo transfer and oocyte yield

As presented in Table 2, of the 107 women that started ovarian stimulation, 102 (95%) had oocyte retrieval and 70 (65% per started cycle) had an embryo transfer. Fifty-seven women (55% per oocyte retrieval) had 3 or less oocytes retrieved, defining low oocyte yield. AMH predicted low oocyte yield with an AUC of 0.81 (95% CI 0.73–0.89), while AFC provided a statistically significant smaller AUC of 0.68 (95% CI 0.57–0.78), *p* = 0.004 (Fig. 2c and Table 3). Adding both AMH and AFC to the model did not increase the predictive value compared with AMH alone. The optimal AMH cut-off value predicting low oocyte yield was 4.0 pmol/L (specificity: 88% and sensitivity: 54%). Both AMH and AFC modestly predicted embryo transfer (Fig. 2d and Table 3).

### Positive hCG test, ongoing pregnancy, and live birth

Twenty-six women out of 107 (24%) had a positive serum hCG test two weeks after embryo transfer resulting in 15 (14% per started cycle) ongoing pregnancies of which all resulted in a live birth (Table 2). Among women with an AMH level < 4 pmol/L only two out of 35 (5.7%) had an ongoing pregnancy and live birth compared with 13 out of 72 (18%) among women with AMH levels ≥4 pmol/L. The lowest AMH value among women with a positive hCG test was 0.98 pmol/L, and 1.3 pmol/L among women that had a live birth. The results from the ROC curves for the prediction of a positive hCG test and live birth are presented in Fig. 2e +F and Table 3. Both AMH and AFC displayed poor predictive value for pregnancy and live birth, though AMH appeared to perform better than AFC with an AUC = 0.66 (95% CI 0.50–0.82, *p* = 0.047) for predicting live birth. Only when combining AMH and AFC could the tests predict live birth with an AUC of 0.70 (95% CI 0.55–0.85, *p* = 0.01). By univariate logistic regression analysis serum AMH concentrations were marginally positively associated with live birth with an OR of 1.21 (95% CI 1.01–1.46, *p* = 0.04).

## Discussion

The availability of new automated AMH assays have opened for a theoretical opportunity to identify the lower AMH threshold for ovarian stimulation. Ideally, a test of ovarian reserve would be able to predict live birth, and to identify those patients that have very limited or no chance of a live birth. These patients might instead opt for oocyte donation avoiding expensive futile IVF attempts. Patients with undetectable AMH levels below the limit of the former AMH Gen II assay have been shown to have acceptable livebirths rates, indicating that the true lower threshold for ovarian stimulation is below the lower limit of detection [14]. In this study, we assessed the ability of AMH measured with the new Elecsys® AMH assay to predict failure to reach the classical criteria for hCG triggering (at least 3 follicles of > 17 mm at day of hCG administration) and to predict cycle cancellation due to complete lack of follicular development during maximal ovarian stimulation for IVF/ICSI.

Our data suggests that an AMH cut-off value of 4 pmol/L predicts failure to reach the classical criteria for hCG triggering with a specificity of 89% (FPR of 11%), and a sensitivity of 53% (FNR of 47%). Hence, among women with AMH < 4 pmol/L, approximately 9 out of 10 would not reach the classical hCG criteria. The low sensitivity and high FNR indicates that almost half of the women not reaching the classical hCG criteria had AMH ≥ 4 pmol/L. However, when establishing the lower AMH threshold for ovarian stimulation, a cut-off value associated with a low FPR (high specificity) would be preferred, even if this would imply a reduced sensitivity, as the results may have wide implications for couples starting ART. In this dataset, a threshold of 4 pmol/L also appeared to provide the best prognostic value for predicting low oocyte yield (≤ 3 oocytes), embryo transfer, pregnancy and live birth. The live birth rate per cycle among women with pre-treatment AMH < 4 pmol/L was lower compared to women with AMH ≥ 4 pmol/l, however this did not reach statistical significance (5.7% vs 18%, *p* = 0.08) probably due to sample size. Even though one baseline AMH measurement does not seem sufficient for withholding IVF/ICSI, as also levels below this cut-off value were compatible with a live birth, the cut-off value of AMH < 4 pmol/L does offer some prognostic value in a population of women with limited ovarian reserve to counsel couples that the chance of livebirth rate will be less than 10% per cycle. Still, the presented cut-off value, should be externally validated on a different dataset to confirm the obtained sensitivity and specificity. Failure to reach the classical hCG criteria was not significantly associated with a lower live birth rate compared with women that did reach the criteria (10% vs 19%, *p* = 0.17). Thus, failure to reach the classical hCG criteria might not be the best indicator of treatment failure.

No international consensus exists for defining a specific criterium for hCG administration. One of the reasons for a lack of standard, is that the criteria for ovulation induction depend on the population studied. In numerous large international multicenter studies, using either the long agonist [20] or antagonist [21, 22] protocol in the “standard” IVF population, the criterium for hCG administration has been 3 follicles of 17 mm or above. Still, lower criteria for hCG administration have been used in studies specifically dealing with patients predicted to have a low or moderate follicular response. Kolibianakis et al. used 2 follicles of 17 mm or above [23], and in the large international ESPART study hCG could be used for triggering when at least one mature follicle was seen on ultrasound [24]. In our study we investigated the chance of achieving the traditional cut-off of 3 mature follicles, but on the other hand we accepted to administer hCG and do retrieval even with 1 or 2 follicles. The rationale for this is that national data-sets show an increase in pregnancy rates for each additional oocyte from 1 to 3 [25], and as we used maximal stimulation doses of hMG, it was unlikely that a cancellation due to inadequate response would subsequently be improved in another cycle, so we pursued oocyte retrieval even with a single mature follicle.

For the prediction of total lack of follicular development (cycle cancellation), we found that an AMH cut-off value of 1.5 pmol/L provided a high specificity and sensitivity and thus this represents an interesting finding. However, the cut-off value was based on a limited number of patients (*n* = 5) and is therefore not robust enough to apply in a clinical setting but warrants further studies. It should also be noted that live birth occurred in one patient with an AMH level of 1.3 pmol/L.

Both AMH and AFC performed equally in predicting failure to reach the classical hCG criteria, cycle cancellation, positive hCG test and live birth. In this dataset, only in the case of predicting poor oocyte yield did AMH perform statistically better than AFC. A meta-analysis from 2013 by Broer et al. (*n* = 5705) found similar predictive value of AMH and AFC for poor response (defined as oocyte yield < 4 or cycle cancellation) and combining the two tests did not improve the predictive value [3]. However, in concordance with our findings Arce et al. showed that AMH had a stronger correlation with oocyte yield compared with AFC (*n* = 749) [26]. These conflicting findings may be due to considerable inter-observer variation in AFC measurements [27], which is one of the major limitations of AFC for prediction of ovarian response, particularly in multicentre trials [28, 29]. This may also be due to the fact that the doctor is more prone to make an optimistic AFC measurement while doing the ultrasound scan.

The main limitation of this study is the sample size which limits the statistical power especially for total lack of follicular development and live birth associations. Furthermore, patients that had undergone previous cycle attempts were also included in this study. Number of previous ART cycles were included in the logistic regression analysis which demonstrated that increasing number of previous ART cycles reduced the risk of failing to reach the classical hCG criteria. This most likely reflects a selection process. Number of previous ART cycles were included in the multivariate logistic regression analysis, in which it did not appear to affect the association between AMH and failure to reach the classical hCG criteria.

The strengths of the study are the use of the new automated Elecsys® AMH assay, the prospective design and a selected population of women with limited ovarian reserve treated with the same fixed high dose gonadotrophin protocol.

In conclusion, an AMH cut-off value of 4 pmol/L can predict the development of less than three mature follicles and low oocyte yield with a FPR of only 11 and 12%, respectively. The live birth rate for women with AMH < 4 pmol/L was 5.7% per started cycle. Thus, one baseline measurement of AMH is not suitable for excluding patients from IVF treatment, even when using a new sensitive assay. Based on our results counselling can be improved as couples with AMH levels below 4 pmol/L should consider how many futile IVF attempts they will accept before opting for oocyte donation. However, studies with repeated AMH measurements over consecutive cycles and cumulative live birth rates over a full treatment course in women with very low levels of AMH measured with the new AMH assays are needed.

## Abbreviations

AFC: Antral follicle count
AMH: Anti-Müllerian hormone
AUC: Area under the curve
hCG: Human chorionic gonadotrophin
POR: Poor ovarian response
ROC: Receiver operator characteristics
